# Association Between the Use of Systemic Steroids and Ocular Hypertension as a Side Effect in Pediatric Population: A Systematic Review

**DOI:** 10.7759/cureus.42112

**Published:** 2023-07-19

**Authors:** Ujala Mushtaq, Basim Shaman Ameen, Chuhao Nie, Daniel Nechi, Iqra J Mazhar, Mohamed Yasir, Saba Sarfraz, Gandhala Shlaghya, Sri Harsha Narayana, Safeera Khan

**Affiliations:** 1 Research, California Institute of Behavioral Neurosciences & Psychology, Fairfield, USA; 2 Internal Medicine, California Institute of Behavioral Neurosciences & Psychology, Fairfield, USA

**Keywords:** side effects, raised intra-ocular pressure, children, systemic steroids, ocular hypertension (oht)

## Abstract

Steroids are commonly used in children for the treatment of various medical conditions. However, systemic steroids can lead to the development of ocular hypertension (OHT), an increase in intraocular pressure. Limited literature is available on the systemic route of steroid administration in children and the development of this side effect. For literature writing and review, a thorough research was conducted across various platforms, such as PubMed, PubMed Central (PMC), Medline, and Cochrane Database of Systematic Reviews (CDSR). After all the screening processes and quality checks, 12 articles were finalized for review writing. The aim was to explore if OHT development is a common side effect developed in children on systemic steroid use for various medical conditions and if any particular risk factors were present among children that lead to its development. The results indicate that OHT is a common side effect of systemic steroid use in children. Children may or may not present with the symptoms of raised intraocular pressure. The development of OHT occurs within one month of the beginning of the steroid treatment in most of the reviewed studies. Several risk factors associated with developing this side effect were also found. In conclusion, systemic steroid use in children leads to the development of OHT. Awareness among healthcare professionals regarding this potential association is necessary. This information can be used to develop guidelines for serial ocular examinations in children on prolonged systemic steroid use.

## Introduction and background

The discovery of corticosteroids more than six decades ago can be considered one of the most important therapeutic revolutions of the last century. Numerous compounds have been synthesized since then, and their use, alone or in combination with other drugs, is crucial for treating many disorders presenting in childhood that have sometimes been lifesaving [1]. Systemic (oral or parenteral) corticosteroids (e.g., prednisone, prednisolone, methylprednisolone, and dexamethasone) possess potent anti-inflammatory, immunomodulatory, and antineoplastic properties and are integral in the treatment of numerous conditions, including autoimmune diseases, allergic reactions, asthma exacerbations, chronic obstructive pulmonary disease, and select malignancies [2].

However, despite these agents' potentially beneficial clinical effects, such use is also associated with serious risks, especially at high doses for extended periods [2]. Corticosteroids can exert various side effects, including hypertension, hyperglycemia, osteoporosis, and psychological symptoms [3]. Using steroids can lead to significant ocular side effects [4]. Ocular hypertension (OHT) is a well-known side effect of glucocorticoid treatment and has been reported following almost every mode of administration, topically and systemically [5,6,7,8].

Corticosteroids are known as strong intraocular pressure (IOP) inducers that would result in OHT, and if it is of a significant magnitude, remains unrecognized, and is left untreated, then glaucomatous optic neuropathy may ensue [9]. Different mechanisms of IOP rise due to corticosteroid (CS) use have been postulated [10]. The risk of steroid-induced ocular hypertension (SIOH) has primarily been investigated following topical application. It has been shown to occur in approximately 1/3 of adults and 2/3 of children depending on the dose and potency of the steroid and risk factors, such as glaucoma, myopia, and age [5,11,12].

By contrast, the literature on the effects of systemic CS use on IOP in the pediatric population has been very limited and controversial [13]. However, many case reports have shown that children can develop very high IOP and sight-threatening glaucomatous optic neuropathy during treatment with high-dose systemic steroids [13,14,15,16]. Young age is a considerable risk factor for severe SIOH [8], probably due to the immaturity of the trabecular meshwork [17,18].

CS effects on gene expression have been investigated as one approach to understand the role of steroids in IOP elevation [19,20]. By the early 1960s, it seemed clear that both OHT leading to primary open angle glaucoma (POAG) and SIOH were influenced by genetics. Studies attempting to define genetic risk factors were undertaken. However, the scientific tools available then were limited; thus, progress stalled [21]. More than 100 genes have been consistently associated with POAG and/or IOP in recent landmark genome-wide association studies. However, these loci have not yet been evaluated in patients with corticosteroid-induced OHT. Myocilin (MYOC) has been the most extensively studied, and a genetic association with MYOC variants or mutations and corticosteroid-induced OHT has not been identified [22]. Other candidate gene studies have been carried out, including analyses of the glucocorticoid receptor (NR3C1) [23]; however, significant association with corticosteroid-induced OHT was not observed, possibly because of the small sample size and reduced power [24].

Human leukocyte antigen (HLA) complex group 22 gene (HCG22) expression has also been shown to increase after treatment of interleukin-1 and decrease after triamcinolone acetate treatment in cultures of trabecular meshwork cells, also supporting the hypothesis that HCG22 may play a role in corticosteroid-induced OHT. Recent technological developments and advancements in scientific knowledge now allow for more comprehensive studies. The confluence of these advances provides important opportunities to identify specific genetic factors influencing this disorder, forming a basis for genetic screening and avoidance of corticosteroids in patients at risk. If genetic variants confer a high risk for corticosteroid-induced OHT are identified, testing for these variants could be used to personalize treatment choices [24].

The main objective of this systemic review is to explore the potential association between the systemic use of steroids in children with diverse medical conditions and the development of OHT as a consequence of the treatment. The review will specifically focus on the systemic administration of steroids and its link to OHT. In addition to identifying existing guidelines, the review also aims to identify specific patient characteristics related to the diseases for which they receive systemic steroid treatment that may be associated with developing OHT as a side effect.

## Review

Methodology

The systematic review was done following the Preferred Reporting Items for Systematic Review and Meta-analyses (PRISMA) guidelines 2020 [25].

Search Sources and strategy

We used PubMed, PubMed Central (PMC), Medline, and Cochrane Database of Systematic Reviews (CDSR) to review articles of interest. A search strategy for each database was formed and applied to get the relevant articles. We used different combinations of keywords, such as "steroids," "OHT," "children," "side effects," "raised IOP," and "pediatric population," to search through databases. Table 1 shows the databases with the used search strategy and the number of papers identified.

**Table 1:** Keywords/search strategies for different databases MeSH: Medical Subject Headings

Search strategy	Database used	No. of papers identified with filters
Steroids and ocular hypertension and children	PubMed (random search)	103
Raised intraocular pressure and steroids and side effects	PubMed (random search)	2
((“Steroids/adverse effects"[Majr] OR "Steroids/toxicity"[Majr])) AND ("Ocular Hypertension"[Majr])	PubMed (Mesh strategy search)	74
((steroids [Title/Abstract]) AND (ocular hypertension [Title/Abstract])) AND (children [Text Word])	PubMed (advance field Search)	13

Eligibility Criteria

Inclusion criteria: Papers involving the pediatric population being treated with systemic corticosteroids for different diseases and showing OHT/raised IOP because of that treatment were included. We selected literature published in the last 10 years, i.e., from 2013 to 2023. Studies that involved human participants exclusively and published in English were included in this systematic review.

Exclusion criteria: Papers published in languages other than English, including adult population, preexisting eye conditions in children, and different modes of steroid intake except systemic were excluded. Moreover, if full-text articles were not retrieved, papers were excluded.

Selection Process

The relevant articles identified by the above strategies were transferred to the end note, and any duplicate articles were removed. We further screened them through abstracts and titles. We further evaluated the shortlisted articles by examining their full texts. We applied the inclusion and exclusion criteria to those full-text articles. The ones fulfilling the criteria were made a part of the review.

Quality Assessment

The shortlisted articles were subjected to a quality appraisal. Different methods were used according to the study type of the article. For case reports and cross-sectional studies, Joanna Briggs Institute (JBI) grading system was used, whereas, for cohort studies, both the prospective and retrospective Newcastle-Ottawa scale was used. Studies satisfying the quality tool criteria were made a part of the systemic review.

Data Collection Process

After quality checking of all the finalized articles, the results, including primary and secondary outcomes, were extracted using data extraction questionnaires to perform qualitative synthesis.

Results

Search Results

**Figure 1:**
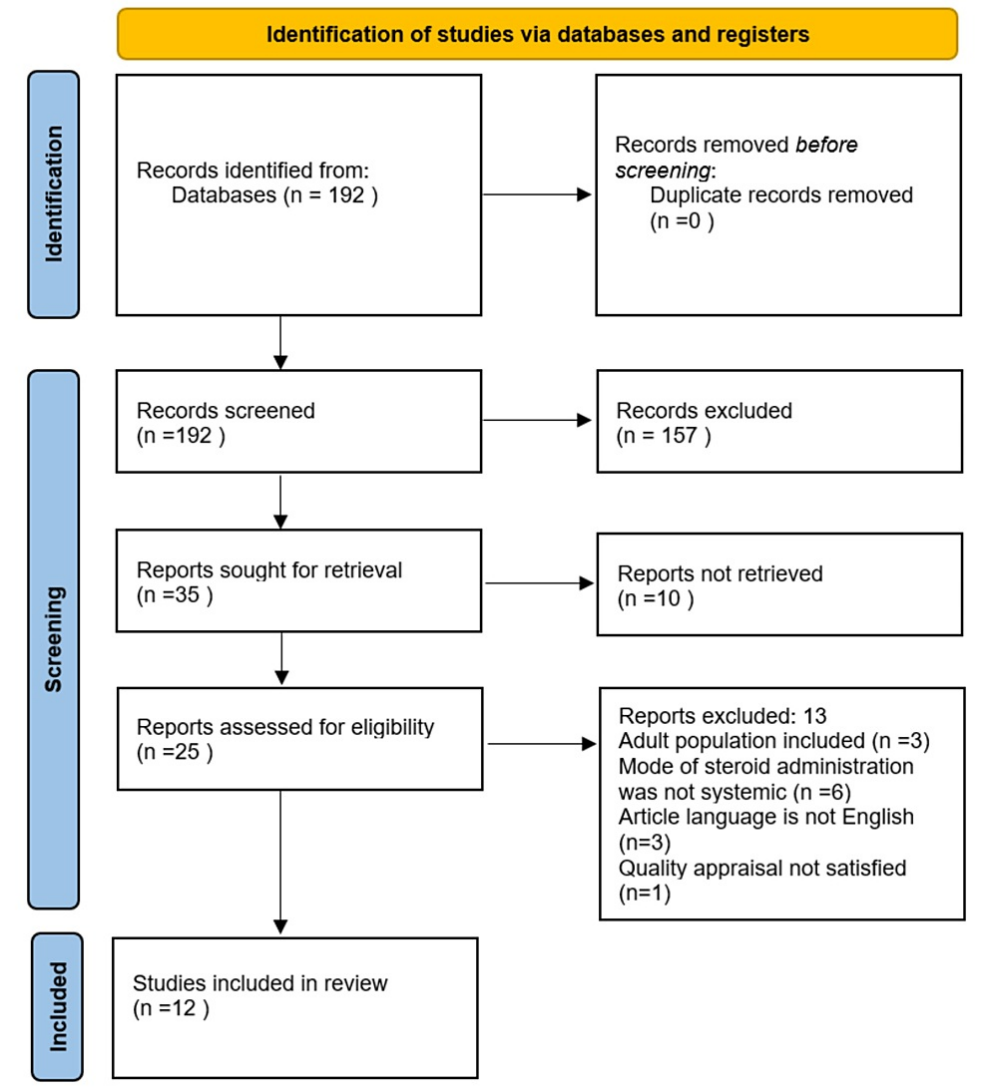
PRISMA flow chart showing the study selection process PRISMA: Preferred Reporting Items for Systematic Review and Meta-Analysis

We identified 192 articles after a thorough literature search through different databases using multiple search strategies. The articles were screened using abstracts, titles, and full texts. The inclusion and exclusion criteria were applied. Thirteen articles were excluded as they did not fulfill the inclusion criteria. Three of them were excluded because they were not published in the English language. The other three included the adult population and, therefore, were not a part of the final articles involved in this systematic review writing. Six were excluded because of different routes of steroid administration, i.e., other than systemic, such as topical and inhalational. One was excluded as it did not satisfy the quality appraisal criteria. In the end, 12 finalized articles were included in the writing of this systemic review. Figure 1 shows the selection process of studies in detail.

The articles were assessed for eligibility using different quality appraisal tools. Table 2 shows the quality appraisal for the cross-sectional study, Table 3 for the case reports, and Table 4 for cohort studies.

**Table 2:** Quality appraisal using JBI for cross-sectional study JBI: Joanna-Briggs Institute

JBI checklist questions								
Study	Q1	Q2	Q3	Q4	Q5	Q6	Q7	Q8
Gaur et al. (2014) [26]	Yes	Yes	Yes	Yes	Unclear	Unclear	Yes	Yes

**Table 3:** Quality appraisal using JBI for case reports JBI: Joanna-Briggs Institute

JBI checklist questions									
Study	Q1	Q2	Q3	Q4	Q5	Q6	Q7	Q8	Q9
Lai et al. (2022) [27]	Yes	Yes	Yes	Yes	Yes	Yes	Yes	Yes	Good
Beverstock et al. (2019) [28]	Unclear	Yes	Yes	Yes	Yes	Yes	Yes	Yes	Good
Tasaki et al. (2021) [29]	Yes	Yes	Yes	Yes	Yes	Yes	Yes	Yes	Good

**Table 4:** Quality appraisal using the Newcastle-Ottawa Scale for cohort studies

Study	Selection	Comparison	Outcome
Sugiyama et al. (2019) [30]	**	*	***
Prasad et al. (2019) [31]	***	*	***
Krag et al. (2021) [32]	**	*	***
Kawaguchi et al. (2014) [33]	***	*	**
De Queiroz Mendonca et al. (2019) [34]	***	*	***
Barzilai Birenboim et al. (2022) [35]	***	*	**
De Queiroz Mendonca et al. (2014) [36]	***	*	**
Hariharan et al. (2021) [37]	**	*	***
Chang et al. (2022)	**	*	**

Outcomes Measured

The primary outcome measured in most of the included studies was to see which ocular complications develop following high-dose steroid treatment for various medical conditions.

The secondary outcomes measured included whether any association exists between the dosage, duration of the steroid use and IOP rise, and any particular risk factors associated with developing OHT in response to steroid usage. A few of them shed light on when ocular complications usually manifest themselves.

Study Characteristics

We reviewed a total of 12 papers that involved 457 participants. Three died, so the total number of participants was 454. Out of the 16 participants, gender distribution was not discussed [32]. Excluding this number, we had a total of 438 patients. Among them, 268 were males and 170 were females. Out of 12, nine studies are observational. Out of nine, two are retrospective cohort studies [30,33], five are prospective cohort studies [31,32,34,35,36], and one is both a retrospective and prospective cohort study [37]. Three studies out of 12 are case reports [27,28,29], and one is cross-sectional [26]. All studies involved pediatric populations being treated via the systemic route of steroid administration for prolonged periods for various medical conditions. The medical conditions discussed for this purpose in the selected studies included hematological malignancies, such as acute lymphoblastic leukemia (ALL), nephrotic syndrome, and autoimmune hepatitis. One case report regarding juvenile idiopathic arthritis is also included [28]. All included studies show prolonged steroid use followed by significantly high IOP/OHT development. The following studies also compared the dosage and duration of steroid use with IOP rise to see if any significant association exists between them [26,27,30,31,32,33,36,37]. The specific duration during treatment with steroids after which changes in IOP were detected is also discussed. Table 5 shows important characteristics of all the finalized studies included in the review

**Table 5:** Summary table of the studies OHT: ocular hypertension; ALL: acute lymphoblastic leukemia; PSSC: posterior subcapsular cataract; I/V: intravenous; IOP: intraocular pressure; SIOH: steroid-induced ocular hypertension

Authors and year of publication	Type of study	Purpose of study	No. of participants	Intervention studied	Results	Conclusions
Gaur et al. (2014) [26]	Cross-sectional study	To determine which type of ocular complications develop in children suffering from nephrotic syndrome who are on long-term oral steroid treatment. Also, to assess if an association exists between the dose and duration of steroids to the development of ocular complications.	82	Long-term oral steroids	Twenty-two patients developed PSCC, nine developed raised IOP, and one developed both.	Ocular complications, such as PSCC and raised IOP, developed in children on long-term oral steroids for nephrotic syndrome. No association was found between the complications' dose, duration, and development.
Lai et al. (2022) [27]	Case report	To report the development of very high IOP in a nine-year-old child following treatment for ALL.	1	Steroids during the maintenance phase of chemotherapy		Patients on ALL chemotherapy should undergo regular eye examinations due to the tendency to develop OHT.
Beverstock et al. (2019) [28]	Case report	To report raised IOP in a girl following I/V methylprednisolone given to treat acute flare of juvenile arthritis.	1	Two doses of I/V methylprednisolone		Patients tend to develop high IOP after systemic steroids, and children may remain asymptomatic or even present with only minimal symptoms. Hence, when on systemic steroids, their IOP should be measured routinely to diagnose this condition early.
Tasaki et al. (2021) [29]	Case report	To report the development of very high IOP in a six-year-old child due to systemic steroid use in treating ALL and chemotherapy.	1	Oral dexamethasone and chemotherapy for ALL		All phases of chemotherapy in ALL involve high steroid doses; hence, the risk of developing OHT increases further. Therefore, regular monitoring of the eyes is needed.
Sugiyama et al. (2019) [30]	Retrospective cohort study	To measure changes in IOP in patients diagnosed with hematological malignancies while receiving chemotherapy containing standard corticosteroid doses.	15	Chemotherapy with standard corticosteroid doses	Thirteen patients developed raised IOP, whereas two did not. IOP was significantly raised with the administration of dexamethasone than prednisolone.	A significant number of patients on chemotherapy develop raised IOP. Most patients remained asymptomatic; therefore, periodic IOP measurements should be done at the beginning of chemotherapy.
Prasad et al. (2019) [31]	Prospective cohort study	To determine if IOP raises/changes in patients who are on long-term oral steroids due to autoimmune hepatitis.	33	Long-term oral steroids	Raised IOP was found in 20 patients at one month, eight at three months, and one at a six-month follow-up visit.	Oral steroids-induced OHT is very common in autoimmune hepatitis patients. It is more prominent in children with decompensated liver disease than compensated.
Krag et al. (2021) [32]	Prospective cohort study	To determine steroid-induced changes in ocular pressure in children suffering from different conditions but being treated with high doses of steroids.	16	High dose of systemic steroids	Nine children (56%) developed SIOH.	Steroid usage is associated with OHT development, but most patients were asymptomatic. No relation between the dose of steroid and duration was observed.
Kawaguchi et al. (2014) [33]	Retrospective cohort study	To determine the frequency and timing of OHT in children of nephrotic syndrome being treated with steroids after the first episode and relapse.	26	Oral steroids (prednisolone)	Eight patients (30%) developed raised IOP and were treated for OHT. Nine patients experienced similar OHT at relapse too.	A significant association exists between OHT occurrence with the use of steroids in nephrotic syndrome patients on the first episode. Also, when previously treated patients relapse, they tend to develop this OHT again. It develops during the initial days of treatment.
De Queiroz Mendonca et al. (2019) [34]	Prospective cohort study	To see which ocular manifestations develop in acute leukemia patients because of their treatment and the factors strongly associated with developing these ocular findings/associations.	67	ALL protocols 1999 and 2009	Thirty-seven patients had a normal ocular examination, whereas 18 patients had ocular manifestations, among which OHT was more frequently found than retinal hemorrhages.	Patients suffering from ALL develop ocular manifestations due to treatment. Most commonly, they develop OHT. A strong association exists between OHT development with ALL 1999 protocol in the group with a high risk of relapse rate.
Barzilai Birenboim et al. (2022) [35]	Prospective cohort study	To determine IOP changes that developed in children newly diagnosed with ALL during their first month of induction therapy with high doses of glucocorticoids.	90	Induction therapy for ALL with high-dose glucocorticoids	Raised IOP was identified in 71% of the cohort (64 patients). Overall, 13 children had to be given OHT reduction therapy.	Significant levels of IOP leading to ocular hypertension are found in the patients of ALL treated with high-dose steroids.
De Queiroz Mendonca (et al.) 2014 [36]	Prospective cohort study	Evaluate changes in IOP in children treated with steroids for ALL & non-Hodgkin lymphoma at different days of treatment therapy.	15	Steroids containing therapy for ALL and non-Hodgkin lymphoma	Two cases of OHT were found because of treatment with steroids for these conditions.	Statistically significant rise in IOP is observed on different days of treatment.
Hariharan et al. (2021) [37]	Prospective + retrospective cohort study	To determine the incidence and risk factors for ocular complications in children on long-term oral steroids for nephrotic syndrome.	110	Oral steroids	Twenty children developed bilateral cataracts; 10 developed raised IOP, and one developed hypertensive retinopathy.	A high incidence of cataracts and raised IOP is found in patients of nephrotic syndrome treated with steroids. Cumulative dosage of steroids, age of onset of nephrotic syndrome, and duration of steroid intake are strongly associated with developing the above complications.

Discussion

Raised IOP due to using steroids is called OHT. If this pressure is too high or present for a long time, it can lead to severe ocular damage. Despite the side effects of steroids, we must use them in certain conditions, such as leukemia, nephrotic syndrome, transplant of organs, inflammatory bowel diseases, and arthritis, as their beneficial effects outweigh the harmful effects of these conditions in the long run.

Steroid Responsiveness, Ocular Complications, and Detection

The literature regarding systemic steroid use in children and its complications is very little. In this review, all the included study patients were using systemic steroids. The conditions for which systemic steroids were used in the reviewed studies included hematological malignancies (n=6), nephrotic syndrome (n=3), and other conditions (n=3), such as autoimmune hepatitis and juvenile idiopathic arthritis Every patient's ocular response to steroids is different. 

Steroid responders can be divided into low, intermediate, and high responders. Becker used the final IOP to grade steroid responders; thus, lower than 21 mmHg was graded as low, 21-30 mmHg as intermediate, and above 30 mmHg as high. Meanwhile, Armaly used the elevation of IOP to rank steroid responders, in which low was below 6 mmHg, intermediate as 6-15 mmHg, and high as above 15 mmHg. The study done by Krag et al. reported that two children (12%) were high responders, seven (44%) were low responders, and the other seven (44%) were high responders [32]. In a few of the studies, each reported a single patient that was categorized as a high responder [5,13,14]. The most common ocular complication patient suffered from during prolonged steroid treatment was OHT.

Out of the 454 patients, 156 developed this complication. The duration at which it was detected was different for different studies. Two studies demonstrated the emergence of the aforementioned complication within a month following treatment [31,32]. One of the research showed maximum IOP at eight days of treatment [27], while Beverstock described the immediate development of raised IOP in his case report [28]. One of the research reported that these complications can be detected as early as six months into the treatment [26]. Kawaguchi et al. demonstrated these to occur within the first week of treatment [33]. The remaining did not discuss specific timing related to the beginning of the complications. Based on the reviewed studies, it can be inferred that a significant factor in ocular complications, such as OHT, is a heightened response to steroid treatment. This phenomenon is observed more frequently in patients who exhibited high responsiveness to steroids than those who were low or non-responders. It can be concluded that ocular pressure changes emerge within the initial month of treatment, underscoring the importance of immediate detection and intervention. Failure to address these changes promptly may result in potential vision problems going unnoticed and causing future complications.

Symptomatic vs. Asymptomatic Presentation

Adults with raised IOP present with some very specific symptoms, such as photophobia, persistent headache, and visual changes. These typical symptoms of raised pressure are not found in children. They can either present with atypical symptoms or may be asymptomatic throughout unless found out for some other reason. The study by Lai et al. showed bilateral blurry halo vision and mild intermittent headache [27]. Takasi’s study reported headaches as a raised IOP symptom [29]. Another study by Prasad et al. mentioned blurring of vision, eye pain, redness, lacrimation, and colored halos [31]. In their study, Sugiyama et al. reported photophobia and headache as prominent symptoms [30]. In one of the studies, patients were observed to exhibit unconventional symptoms, such as lethargy and excessive drowsiness, compared to their usual state [28]. Two of the studies reported patients with raised ocular pressures, but they did not develop any symptoms of it [32,35].

In some cases, even if symptoms were present, children could not communicate. Due to this extreme variation in symptoms, we must be vigilant to catch this complication early in development. Based on the collective findings of the studies, it can be inferred that ocular complications manifest in various ways. The range of symptoms observed is extensive, ranging from mild indications, such as watering and redness, to severe ones, such as vision loss and sensitivity to light (photophobia). Regarding children experiencing prolonged steroid pressure changes, predicting which specific symptoms will be present is challenging. Therefore, it is crucial to maintain a high level of suspicion to detect these changes. As demonstrated in the reviewed studies, children often display no symptoms or vague symptoms that do not typically correlate with ocular pressure changes.

Risk Factors for the Development of OHT With Steroid Usage

Multiple risk factor associations with the use of systemic steroids and the development of OHT as a side effect were found in the studies. One of them is a young age. Krag’s study reported that steroid responders were significantly young [32]. Lia’s research also concluded that the ALL patient in his study who was a high responder belonged to the younger age group [27]. It was thought that young age in ALL patients might be associated with the immature structure of the trabecular meshwork. Some of the studies under review concluded that younger age is an important risk factor [29,30,34]. Sugiyama et al. also pointed out that younger patients with ALL aged <nine years treated with oral dexamethasone had higher IOP than those with >10 years who were similarly treated [30]. It can be because of continuous dexamethasone exposure or the difference in the degree of ER among younger and older patients.

The relationship between increased steroids and raised IOP was also noted. One of the studies concluded that with higher doses of dexamethasone, higher elevation in IOP was noted [27]. Hariharan noted this association but not with IOP [37]. According to his incidence studies of ocular complications due to steroid use, increased steroid doses led to more cataract formation rather than raised ocular pressures. Sugiyama’s research also demonstrated a similar relation but with decreasing dosages [30]. His study revealed that by decreasing the doses of steroids, the ocular pressure decreased accordingly. De Queiroz Mendonca et al. also concluded that high steroids are associated with higher IOP values [36]. Another study also reported similar findings of raised ocular pressures with increased dosage and decreased pressures in the eye with a decrease in dosage of steroids [33]. Studies conducted by Krag, Prasad, and Gaur et al. reported no such association [32,31,26].

Among all the studies in this systematic review, the following included patients treated primarily with systemic prednisolone (type of steroid) [26,28,30,31,32,33,37]. Meanwhile, in others, all patients were treated with only dexamethasone (another type of steroid) [27,29,30]. Some of the studies included patients who were given prednisolone and dexamethasone during the treatment [30,34,35,36]. However, it was seen that with either type of steroid in use, the IOP tends to increase. However, in comparison to prednisolone, oral dexamethasone causes a more significant increase in IOP. Takasi’s study concluded higher ocular hypertensive response with oral dexamethasone in ALL patients [29]. They were thought to be caused because they can better penetrate the central nervous system (CNS) and have a longer half-life, thus making them an ideal choice of steroid for use ALL patients. Research conducted by Sugiyama stated that three out of four patients who were given prednisolone and dexamethasone developed significantly high IOP only with dexamethasone [30]. During each chemotherapy course in which particular steroids were given, dexamethasone caused higher ocular pressures than prednisolone. One of the reasons mentioned by Sugiyama behind it was that dexamethasone increases the secretory protein load of extracellular matrix proteins in the endoplasmic reticulum of trabecular meshwork cells, thereby including chronic endoplasmic reticulum (ER) stress and promoting OHT via the transforming growth factor 𝛽2 signaling [30]. De Queiroz Mendonca’s study reported two patients with elevated ocular pressures, and the one with dexamethasone usage had more elevated ocular pressure than the one treated with prednisolone [36].

Barzilai’s research also concluded that dexamethasone is a significant risk factor for OHT [35]. He also identified other risk factors, particularly for ALL patients, that led to significant ocular pressure changes during treatment with systemic steroids. These were ocular hypertension at the time of diagnosis of ALL, children with high WBC count at ALL diagnosis, elevated BMI percentile, and a family history of glaucoma. De Queiroz Mendonca also pointed out that children suffering from ALL who developed ocular hypertension because of systemic steroid treatment were commonly those who were treated with ALL-99 protocol, had a higher risk of relapse, presented with CNS infiltration at one examination and B cell immunophenotype, and of younger age group [34].

It can be deduced that several notable risk factors are associated with the prolonged use of steroids, leading to the development of OHT. One prominent and consistent finding across most studies is the frequent use of dexamethasone, a specific type of steroid, which consistently resulted in the highest ocular pressure among all treated patients. Furthermore, another prevalent observation identified in nearly every study is that younger individuals are more susceptible to experiencing elevated IOP when subjected to steroid treatment.

Limitations of the Study

This literature review has some limitations also. All the studies included in this review were observational, i.e., cohort, case reports, and cross-sectional studies. None of the clinical trials or other systemic reviews were included. The study population was small. The IOP was not measured using a Goldmann applanation tonometer, a gold standard for this purpose. Different types of tonometers were used in the reviewed studies, which might lead to different baseline values for the comparison of IOP.

## Conclusions

Our systematic review sheds light on the possible association between systemic steroid use and OHT and the possible association between systemic steroid use and OHT as a side effect in the pediatric population. The findings suggest that most children who receive systemic steroids experience OHT as a common side effect, although symptoms can be atypical or absent. Several risks were identified for this association, such as young age, oral dexamethasone use, and increased dosage of steroid-led response. However, there are no clear guidelines on routine ocular checkups for children on prolonged steroid therapy for systemic illnesses. Our review emphasizes the need for policymakers and healthcare providers to implement appropriate policies to monitor ocular health in children receiving systemic steroids. Increased awareness among healthcare providers about the potential ocular side effects of steroid use in children is crucial to improve care quality. Furthermore, additional research is necessary to understand this phenomena's genetic associations better and develop more effective treatment approaches.
